# Spectrum of alpha-globin gene mutations among premarital Baluch couples in southeastern Iran

**Published:** 2015-07-01

**Authors:** Ebrahim Miri-Moghaddam, Abass Nikravesh, Negin Gasemzadeh, Mahin Badaksh, Nahid Rakhshi

**Affiliations:** 1 Genetics of Non-communicable Disease Research Center, Zahedan University of Medical Sciences, Zahedan-Iran; 2 Department of Genetics, Zahedan University of Medical Sciences, Zahedan-Iran; 3 Department of Molecular Sciences, Faculty of Medicine, North Khorasan University of Medical Sciences, Bojnurd-Iran; 4 Esfarayen Faculty of Medical Sciences, Esfarayen, Iran; 5 Department of Biology, Faculty of Basic Sciences, Zabol University, Zabol-Iran; 6 Department of Midwifery, Faculty of Nursing and Midwifery, Zabol University of Medical Sciences, Zabol-Iran; 7 Department of Nursing and Midwifery, Bojnourd branch, Islamic Azad University, Bojnourd, Iran

**Keywords:** Alpha thalassemia, prevalence, mutation, Baluch, Iran

## Abstract

**Background:** Alpha thalassemia (α-thal) is one of the most common hemoglobinopathies worldwide. The aim of this study was to investigate the spectrum of α-thal mutations among premarital Baluch couples in southeastern Iran.

**Subjects and Methods: **We assessed 1215 individuals by multiplex gap polymerase chain reaction (gap-PCR) and amplification refractory mutation system (ARMS-PCR).

**Results: **Of the 1215 participants with mean age of 23±5.7 years, 62.3% lived in urban areas, and the rate of consanguineous marriage was 68.1%. Five mutations were identified, the most frequent one was –*α*^3.7^ (rightward) with a frequency of 76.5%, followed by *α*^−5 nt^ (16.8%), α2/ Codon 19(-G) (4%), –*α*^4.2^ (leftward)(2.4%), – –MED (0.3%) among mutated alleles of the α -globin gene.

**Conclusion** **:** Knowing the alpha-genotype is helpful for genetic counseling, microcytic anemia discrimination and hemoglobinopathy prevention.

## Introduction

 Alpha-thalassemia (α-thal) is one of the most common inherited single gene disorders in humans resulting decrease or absence in production of the alpha-chain of hemoglobin. The α-globin gene cluster is located on the short arm of chromosome 16 (16p 13.3), which includes α2, α1 and embroyonic zeta globin genes.^   1 ^ The severity of the clinical phenotype of α-thal is diverse and depends on the copy number of α gene affected and the type of deletion or non-deletion mutation occurred.

The most clinical phenotype of individuals with α-thal is mild and recognized during a routine complete blood count. α-thal mutations occur in almost all populations,  ^2^^,^^3^  while the carrier frequency of α-thal gene varies between 1% to 98% throughout the world.^   4 ^ Sistan and Baluchistan province in the south-east of Iran, with a population of approximately 2.7 million people (in all about 60% of people are Baluch), is located in the sub-tropical region where malaria is reported.^        5 ^ Studies show that α-thal carriers have been selected and relatively protected in an endemic malarial area.^6^^,^   7^,^^8^ 

α-thal is a heterogeneously inherited disorder of α-globin synthesis that more than 128 different molecular defects have so far been described.^   9 ^ Iran is a country with multiethnic groups, different distributions of α -globin mutations are expected in various populations. Therefore, it is necessary to be determined of the frequency of α-thal mutations and its effects on hematological indices in each ethnic population. Thus, knowing the prevalence of mutations could help in screening for hemoglobinopathies and anemia treatment.

The aim of this study was to determine the spectrum of α-thal mutations in Balouchi couples referred to pre-marriage tests.

## SUBJECTS AND METHODS

 This study was carried out in the hematology departments of reference laboratories in Zahedan and Zabol; two cities in the Sistan and Baluchistan province. After the written consent was obtained, blood samples were taken in EDTA-contained tubes. The study protocol was approved by the Ethics Committee of the Research Deputy of Zahedan University of Medical Sciences. 

Red blood cell (RBC) indices were determined using automated cell counter (Sysmex K1000; Sysmex, Tokyo, Japan). The Hb separation was investigated by alkaline electrophoresis on cellulose acetate strips at pH 8.5 and evaluation of HbA2 was performed using micro column chromatography.^        10^

All study participants possessed MCV<83fL, MCH<28pg and HbA2<3.5%. Genomic DNA was extracted from peripheral blood leukocytes by the phenol-chloroform extraction method.   11  The most common deleted mutations in the α-globin genes [–α^3.7^ (rightward), –*α*^4.2^ (leftward), – –MED and – (α) 20.5 kb] were evaluated using gap-PCR^   12 ^ and two non-deleted α-thal mutations [ *α*^−5 nt ^and codon 19 (–G)] by ARMS-PCR. Statistical analyses were performed using ANOVA to compare the means of haematological indices measured within the *α- *thal phenotypic groups. The post-hoc range tests of LSD were used to determine which parametric means differed by pair-wise comparison of different phenotypes. All data were analyzed using SPSS software version 16.

## Results

 We evaluated 1215 Baluchis who were referred for pre-marriage thalassemia screening tests by health care centers. The age ranged from 13 to 57 years and the mean age was 25.1±6.1 and 21.3±4.8 years for men and women, respectively. A total of 62.3% of participants came from urban and 37.7% from rural areas, the ratio of consanguineous marriages (first or second cousin) to non-consanguineous marriages was 68.1:31.9. 

Five different deletion mutations in α -globin genes were identified in which -α^3.7 ^was the most frequent with 76.6%, followed by non-deletion mutation codon IVS-I, -5 nt/α with 16.6%, and the α2/codon 19 (-G) with 4% which constitute 97.2% of all mutated alleles of α -globin gene. The allele frequencies of α-globin gene mutations in Baluchi population of Sistan and Baluchistan province are shown in Table 1. The correlations between α-globin genotypes and values of hematological indices are listed in Table 2.

## Discussion

 Thalassemias are the most common monogenic disorders that affect hematological indices. A large number of participants with reduced MCV, MCH, normal HbA_2_ and F levels were referred to health care centers and clinics as suspicious cases of a-thal carriers. Our results are consistent with Adorno et al.^   13 ^ and Souza et al.^   14 ^ which show that RBC count in α-thal subjects increased and Hb, Hct, MCV and MCH decreased significantly in comparison to individuals with normal α-genes (αα/αα).

**Table 1:** Allele frequency of α-globin gene mutation in premarital Baluch couples in Sistan and Bluchistan province, Iran

**Mutation**	**Number**	**%**
**- α** ^3.7^	245	76.6
**α** ^-5nt^ ** (codon IVS-I, -5 nt/α)**	53	16.6
**α** ^CD19^ ** (α2/ Codon 19(-G))**	13	4
**- α** ^4.2^	8	2.5
**- -** **Med**	1	0.3
**Total**	340	100

**Table 2:** Distribution of α-globin gene mutations in premarital Baluch couples in Sistan and Bluchistan province with mean and standard deviation of RBC indices

**Genotypes**	**N**	**%**	**RBC** **(x10** ^6^ **/mm** ^3^ **)**	**Hb** ** (g/dl)**	**Hct ** **(%)**	**MCV** **(fl)**	**MCH ** **(pg)**
**αα /- α** ^3.7^	172	66	5.6 ± 0.67	13.2 ± 1.9	42 ± 5.3	75.3 ± 5.8	23.8 ± 2.7
**- α** ^3.7^ **/- ** **α** ^3.7^	30	11.5	6 ± 0.83	12.5 ± 1.3	42.2 ± 5.2	69.9 ± 3.3	20.8 ± 1.7
**αα / αα** ^-5nt^	14	5.4	6.2 ± 0.71	13 ± 1.1	43.7 ± 4.2	73.8 ± 4.1	22.4 ± 2.1
**- α** ^3.7^ **/** ** αα** ^-5nt^	13	5	6.05 ± 0.77	13 ± 1.1	42.8 ± 4.9	70.5 ± 6.3	21.5 ± 2.8
**αα** ^-5nt^ **/** ** αα** ^-5nt^	13	5	5.8 ± 0.62	12.9 ± 1.8	41.3 ± 5.5	73 ± 4.6	22.6 ± 2
**αα/αα** ^CD19^	4	1.5	5.9 ± 0.65	12.5 ± 1.5	42.3 ± 4.6	70.9 ± 3.5	21 ± 2.1
**- ** **α** ^3.7^ **/ ** **αα** ^CD19^	3	1.15	6.3 ± 0.79	13.9 ± 1.5	45.5 ± 5.8	71.7 ± 3.6	22.1 ± 3.2
**- α** ^4.2^ **/** **- α** ^3.7^	3	1.15	6.4 ± 0.38	13.9 ± 1	46.1 ± 2.8	71.7	21.6 ± 1
**αα** **/** **- α** ^4.2^	3	1.15	5.8 ± 0.35	14.6 ± 1.5	44.5 ± 2.7	75.8 ± 1.2	24.8 ± 1.3
**αα** ^CD19^ **/** ** αα** ^-5nt^	2	0.75	5.4 ± 1.22	11.7 ± 2.4	38.5 ± 8.2	71 ± 0.8	21.6 ± 0.4
**αα** ^CD19^ **/- ** **α** ^4.2^	2	0.75	6 ± 0.49	12.4 ± 0.7	43 ± 3.8	71.2 ± 0.5	20.5 ± 5
**αα** ^CD19^ **/** ** αα** ^CD19^	1	0.325	6.7	14.4	42.1	69.1	21.5
**αα/--** **Med**	1	0.325	6.6	11.8	34.6	68.8	20.5
**Total**	261	100.0	5.1 ± 0.6	13.8 ± 1.6	42.5 ± 4.4	82.5 ± 6.29	26.9 ± 2.8

This alteration in α –thal mutations carriers were to be expected since the unbalanced globin synthesis in α-thal led to a 30-35% decrease in the amount of Hb of the RBC content, causing hypochromic and microcytic anemia.^15^

 The results showed that -α^3.7 ^was the most common mutation (76.6%) followed by *α*^−5 nt^ (16.6%) and α2/codon 19(-G) with 4% among mutated alleles of the α -globin gene. -α^3.7^ and *α*^−5 nt^ and the polyadenylation signal (poly A1; AATAAA>AATAAG) were the three most common mutations (71.7%, 7.0% and 4.2%, respectively)^16^ in microcytic hypochromic anemia cases in two provinces of southern Iran: Fars & Kohkeloye and Bouyer Ahmad. In Khuzestan province, in southwest Iran, -α^3.7^ with 62.6% was the most frequent identified variant. Compound heterozygous mutations were also reported significantly in this region.^17^ In Mazanderan province, northern Iran, -α^3.7^ (44.9%), polyadenylation signal (poly A2; AATAAA>AATGAA) (18.2%), –*α*^4.2^ (9.1%), *α*^−5 nt ^(6.5%), - - (MED) (4.3%), and α2/codon 19(-G) (4%) were the most frequent α -thal mutations in hypochromic microcytic cases.^18^ Evaluation of 103 hypochromic and microcytic anemia patients in Gilan province, northern Iran, showed α-globin mutations in 94 (91.3%) patients, while the most prevalent of α-thal alleles was -α^3.7^ (42.5%), followed by the polyadenylation signal (poly A2); AATAAA>AATGAA) (12.4%), Hb Constant Spring (10.6%), --(MED) (8.8%), *α*^−5 nt^ (7.1%), –*α*^4.2^ (4.4%) and polyadenylation signal (poly A1; AATAAA>AATAAG) (3.5%).^19^ Results from the study on individuals with decreased RBC indices and normal Hb A_2_ in Kerman province (close neighbor to Sistan and Baluchistan) showed that -α^3.7^with 83.8% , followed by α2/codon 19(-G) with 5.7% and *α*^−5 nt ^with 4.2% were the common mutations.^20^

Unlike β-thal, in which non-deletion mutations predominate, more than 95% of recognized α -thal involves deletion of 1 or both α-globin genes from chromosome 16. Reciprocal recombination between Z boxes produces a chromosome with -α^3.7 ^deletion containing only one functional α gene (-α^3.7^ or rightward deletion). Likewise, a reciprocal recombination between mispaired homologous X boxes produces a 4.2-kb deletion (-α^ 4.2^ leftward deletion).^21^ The -α^3.7^ and –*α*^4.2 ^deletions are the most common alpha^+^ α -thal deletion defects. In general, the -α^3.7 ^single gene deletion has a global distribution among all ethnic groups, especially prevalent in most tropical and subtropical populations studied.^12^

## CONCLUSION

 Alpha -thal mutation spectrum among Baluch ethnic are similar to those reported from south neighboring countries and other parts of Iran; however these similarities with the southern provinces are stronger in comparison to northern and western Iran. Knowing the alpha-genotype is useful for genetic counseling and prenatal diagnosis in couples who may present reduced RBC indices with normal HbA2 levels, discrimination the anemia and prevention of hemoglobinopathies.
